# Glenohumeral relationships at different angles of abduction

**DOI:** 10.1007/s00276-014-1315-5

**Published:** 2014-05-27

**Authors:** Hiroaki Inui, Hiroshi Tanaka, Katsuya Nobuhara

**Affiliations:** Nobuhara Hospital and Institute of Biomechanics, 720 Haze, Issaicho, Tatsunoshi, Hyogo 679-4017 Japan

**Keywords:** Shoulder, Glenohumeral joint, Abduction, Function, MRI

## Abstract

**Purpose:**

The objective of this study was to clarify the relationships among anatomical landmarks of the glenohumeral joint at different angles of abduction.

**Methods:**

Fifteen volunteers (ten men, five women; mean age 29 years) were enrolled in this study. Images of externally and internally rotated positions at 45°, 90°, and 135° of abduction in the plane 30° anterior to the trunk were taken using an open magnetic resonance imaging system. Landmarks including the glenoidal long axis with its center, bicipital groove, center of the head, and humeral shaft axis were determined. Using a line set on the surface of the head in the plane parallel to the humeral axis (including the head center and bicipital groove with its parallel and perpendicular lines), the glenoid location and rotational relationships were investigated in each position.

**Results:**

The average angles of axial rotation were 48° ± 27° at 45º of abduction, 71° ± 20° at 90° of abduction, and 40° ± 27° at 135° of abduction. The trajectories of the glenoid center primarily extended over the anterior portion of the humeral head at 45° of abduction and over the posterior portion at 90° of abduction, while those at 135° of abduction were localized on a small upper portion of the head.

**Conclusions:**

The glenohumeral relationships demonstrated that arm abduction might influence shoulder function through its effects on the portion of the humeral surface in contact with the glenoid during rotation and the resultant changes in the glenohumeral relationships.

## Introduction

The shoulder has a wide range of motion [3, 12, 13]. The portion of the glenoid supporting the humeral head and the alignment of muscles connecting the bones drastically change within this range of motion. Thus, the function of the shoulder joint that involves holding and moving the arm may change or be influenced by glenohumeral positioning. The range of rotation becomes restricted when the arm is elevated. The insertion of the short rotators approaches the glenoid surface, and the capsule surrounding the head tightens with arm elevation. This suggests that compared with positions in which the arm is lowered, highly elevated arm positions are more suitable for holding rather than moving the arm. The humeral head has some offset [2, 14, 15] and pivots against the glenoid, making glenohumeral relationships difficult to determine based only on arm positioning. Some studies that investigated contact areas of the joint described the glenohumeral relationships during specific rotational movements [1, 7, 18]. However, information on glenohumeral relationships among various anatomical landmarks during arm rotation at different angles of abduction is scarce, and how glenohumeral positioning affects shoulder function remains unclear. In a previous study, we investigated the glenohumeral relationship in maximum elevation and showed that the long axis of the glenoid coincided with a line set on the surface of the head in the plane parallel to the humeral axis that involved the head center and bicipital groove [10]. We hypothesized that the glenohumeral relationships would be clarified using this line set on the humeral surface. The objective of the current study was to elucidate the glenohumeral relationships during rotation at different angles of abduction.

## Materials and methods

Fifteen volunteers (ten men, five women) without symptoms or a history of shoulder disease were enrolled in this study. Their mean age was 29 years (range 21–35 years). All participants provided informed consent. The present study was approved by the IRB of our hospital.

**Fig. 1:**
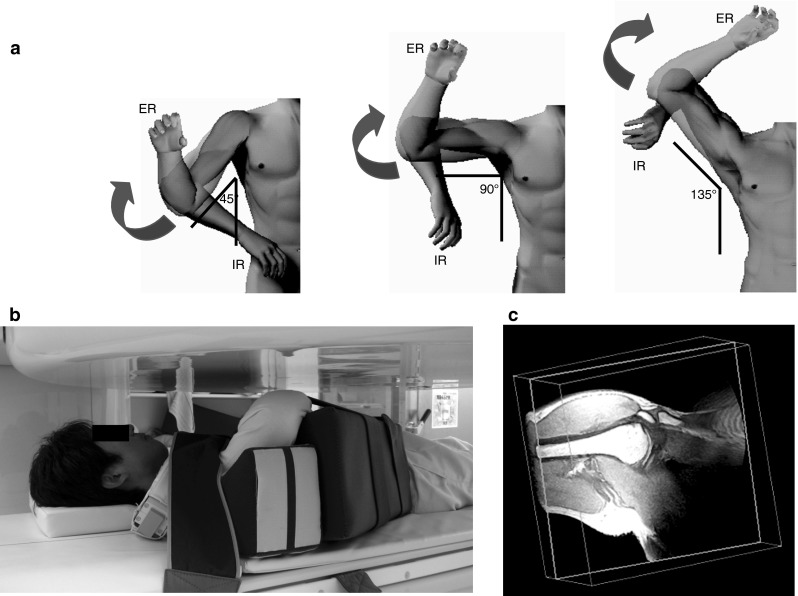
**a** The angle of the upper extremity in relation to the trunk was controlled to determine the six positions. **b** The volunteer was placed in an open magnetic resonance imaging system. The arm position was controlled by a positioning device. **c** A three-dimensional computer-generated magnetic resonance image

The right arm was both maximally externally and internally rotated at abduction angles of 45°, 90°, and 135° in the plane 30° anterior to the trunk. The arm was considered to reach the final position at about 135° of elevation when the humerus was perpendicular to the glenoid and the angle was divided to determine the three elevation angles. The elbow was flexed for relaxation. The amount of pronation or supination of the forearm was not specified. Images of these six positions were obtained using a 0.2-T MRI system (Magnetom Open; Siemens, Munich, Germany) (Fig. 1). Each upper extremity was controlled by a positioning device while maintaining the same relaxed position without disturbing scapular motion. The shoulders were imaged using a three-dimensional (3D) gradient echo (repetition time 56 ms; echo time 25 ms; flip angle 40°) with 2-mm sections. All images were obtained with an 18-cm field of view and a 256 × 192 matrix. Each imaging process required an average of 10 min. All data were transferred to a computer (O2; SGI, Mountain View, CA), and a 3D image of the glenohumeral joint including the proximal part of the humerus was generated using computer software (3D-Virtuoso; Siemens). This software allowed anatomies to be viewed from any angle and provided instant access to 3D information.

Anatomical landmarks such as the glenoidal long axis, glenoid center, humeral head center, and humeral shaft axis were defined as previously described [9]. The point just posterior to the coracoid base on the glenoid rim was defined as the upper rim, and the point just anterior to the lateral border of the scapula was defined as the lower rim. The line connecting these points was defined as the glenoidal long axis. The glenoidal plane was defined as the plane including the glenoidal long axis and was parallel to the line connecting the anterior and posterior rims on a cross-section at the center level of the glenoid.

**Fig. 2:**
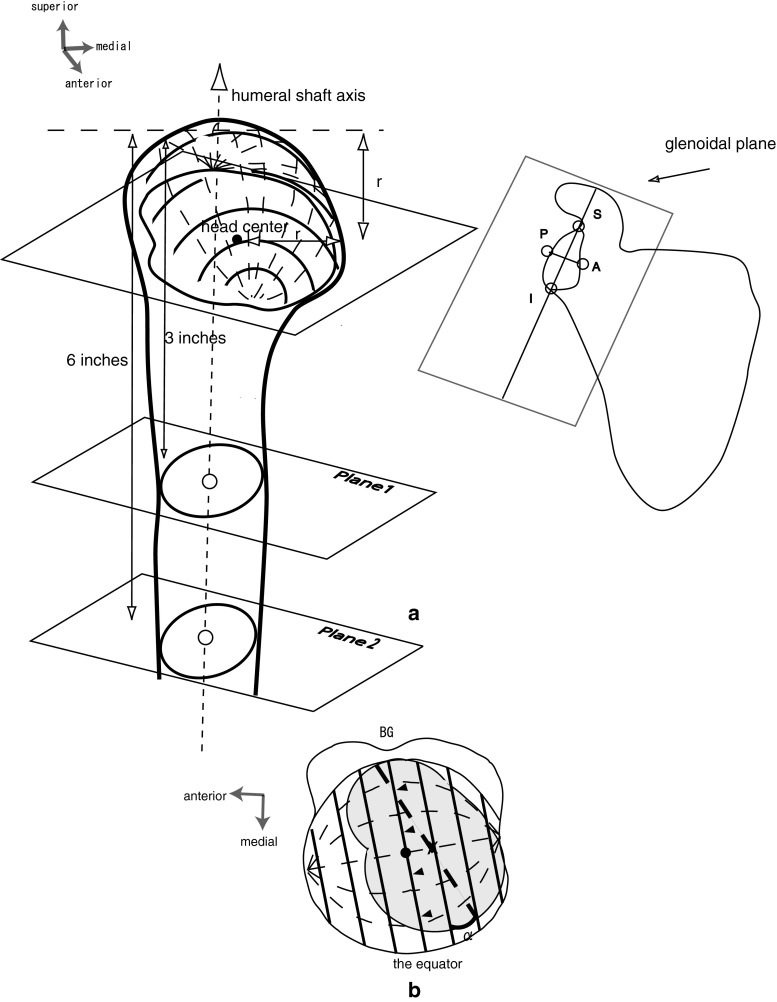
**a** Illustrations showing the anatomical landmarks including the glenoidal long axis (line between the superior and inferior rims), glenoidal transverse axis (line between the anterior and posterior rims), humeral head center, and humeral shaft axis. *A* anterior rim, *P* posterior rim, *S* superior rim, *I* inferior rim, *r* humeral radius. **b** Global diagram set on the surface of the head with the plane including the center of the head (*black dot*) and the bicipital groove, and the parallel planes analogous to the latitudes. The *straight lines* represent circles of latitude, and the *curved broken lines* represent circles of longitude. Rotation (*α*) is referenced to latitude by rotating the globe to align the longitude including the midpoint (*X*) of the glenoidal long axis (*straight broken line*) with the vertical. *BG* bicipital groove

Two cross sections of the humerus were obtained in the plane 1 and the plane 2 at 3 and 6 in. from the proximal end (Fig. 2a). The center of these cross sections of the cortical bone was determined by fitting a circle, and the humeral axis was defined as the line that passed through the center of these circles. Using the data of Iannotti et al. [8], which showed correlations between the size of the glenoid and the radius of the curvature of the humeral head, each humeral radius was calculated as follows: radius (mm) = 24 × length of glenoidal long axis/39 (where 24 is the average head radius and 39 is the average glenoidal long axis). The head was cut in the plane perpendicular to the humeral axis at the radius from the proximal end, and the center was determined by fitting a circle of the same radius. This was regarded as the center of the head. In this plane, the bottom of the bicipital groove was also plotted.

Three parameters including abduction, horizontal abduction, and axial rotation were used to determine joint positioning in each position. The slope of the humeral long axis on the glenoid was determined by measuring the angle relative to the glenoidal long axis (abduction angle) and analyzing the plane of abduction. The latter was shown by the angle of its plane to the glenoid plane (horizontal abduction angle). Axial rotation of the glenohumeral joint was visualized on the computer screen as follows (Fig. 2b). The equator as pointed by arrow heads in Fig. 2b was set on the head surface in the plane parallel to the humeral long axis, including the head center and bicipital groove. Its parallel lines were analogous to the latitudes. The rotation was referenced to the latitude by rotating the globe to align the longitude, including the midpoint of the glenoidal long axis, with the vertical. The angle at which the glenoidal long axis became parallel to the latitude was defined as 0°, and all values in external rotation were defined as positive. The surface of the humeral head was divided into four segments (anterior–superior portion, Zone I; posterior–superior portion, Zone II; anterior–inferior portion, Zone III; and posterior–inferior portion, Zone IV) using the equator and the circle of longitude crossing the top of the head, and the location of the glenoid center was investigated. The glenoid trajectory was determined by connecting the glenoid centers between the internal and external positions at each abduction angle. To confirm the relationships among the anatomical landmarks, the shaft axis and the center of the humerus in each subject were projected orthogonally to the glenoid plane.

Variability and reproducibility were previously reported [10]. Two independent investigators analyzed ten different glenoids by measuring the lengths of the long and transverse axes to determine the interobserver variability. The lengths of these axes in ten glenoids were measured twice by the same person to determine the intraobserver variability. The angles of the plane including the shaft axis and center of the head to the equator on the surface of the head were also analyzed to determine these variabilities with respect to the humerus. The lengths of the long and transverse axes of the glenoid and the angles of the plane including the shaft axis and center of the head to the equator on the surface of the head were 36.0 ± 3.0, 22.5 ± 2.1 mm, and 10.0° ± 8.8°, respectively. Among the ten glenoid and humeral bones, variability and reproducibility were high with a correlation coefficient ranging from 0.73 to 0.98 (Wilcoxon signed-rank tests).

## Results

The angles of abduction, horizontal abduction, and axial rotation in each position are shown in Table 1. The standard deviation in the angle of horizontal abduction in the internally rotated position at 45° of abduction was large, showing the variability in the positioning of the shaft in this position.

**Table 1:** Glenohumeral angle

Position	Abduction	Horizontal abduction	Axial rotation
45° external rotation	20° ± 12°	95° ± 26°	−2° ± 16°
45° internal rotation	23° ± 11°	107° ± 25°	−53° ± 23°
90° external rotation	44° ± 9°	108° ± 11°	8° ± 22°
90° internal rotation	46° ± 9°	113° ± 11°	−70° ± 29°
135° external rotation	83° ± 10°	100° ± 8°	23° ± 17°
135° internal rotation	78° ± 7°	98° ± 11°	−11° ± 26°

**Fig. 3:**
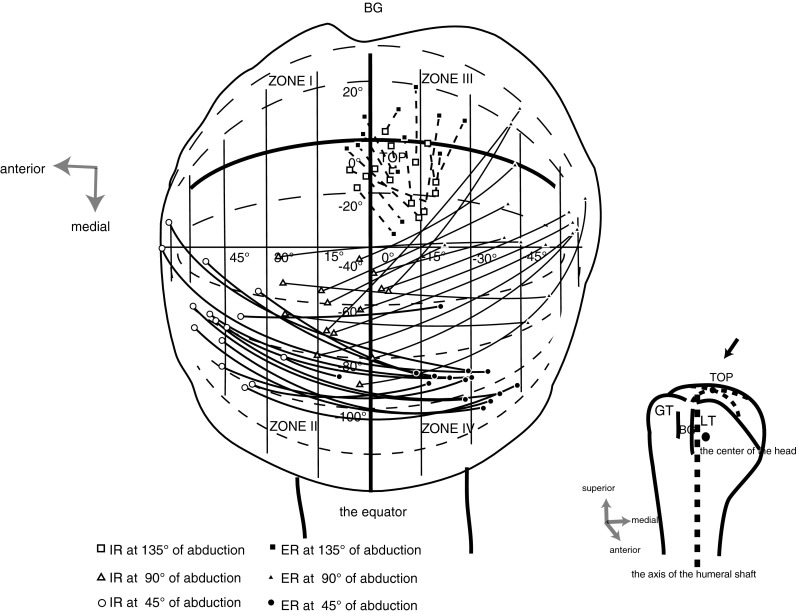
The glenoid trajectories extended largely over the anterior portion of the humeral head at 45° of abduction and over the posterior portion at 90° of abduction. The trajectories at 135° of abduction were localized on a small upper portion of the head

Figure 3 shows that the glenoid trajectories extended primarily over the anterior portion of the humeral head at 45° of abduction and over the posterior portion at 90° of abduction. The trajectories at 135° of abduction were localized on a small upper portion of the head. The lengths of the glenoid trajectories at each abduction angle are compared as the angles in Table 2. The value at 135° of abduction was much smaller than those at lower abductions (paired *t* test: *p* < 0.0001, *p* < 0.0001).

**Table 2:** Glenoid motion during rotation in three abducted positions

Position	Glenoid motion
45°	73° ± 28°^a^
90°	72° ± 28°^b^
135°	22° ± 16°^a,b^

^a,b^ Paired *t* test; *p* < 0.0001

## Discussion

The aim of the current study was to reveal the glenohumeral relationships during rotation at different angles of abduction. Healthy shoulders maintain stability within a wide range of motion. Previous studies on the positioning of the shoulder joint mainly focused on whether the joint is centered in its range of motion [6, 16, 17]. However, the portion of the glenoid supporting the humeral head and the alignment of muscles connecting the bones drastically change within this range of motion. The function of the shoulder joint may change or be influenced by glenohumeral positioning, but information on the location of the glenoid supporting the humeral head and other relationships among the anatomical landmarks during arm rotation at different angles of abduction are scarce.

Several authors have reported the benefits of using an open MRI system to analyze the 3D kinematics of the shoulder joint [5, 10, 11]. An open system allows for investigation of the joint in functional positions without radiation exposure to patients. Another advantage is that the neuromuscular control mechanisms are preserved when the scapula is free to move. Additionally, the relationships among the bony landmarks can be evaluated through trial and error on a computer screen.

In a previous study investigating the rotational alignment of the joint in maximum elevation, we set a line on the surface of the head using the plane parallel to the humeral axis and involving the center of the head and the bicipital groove [10]. The final position was unique in that the glenoid long axis coincided with that line, with its center located at the top on the line. Additionally, the glenoid could be reached to the point regardless of the course of the humerus. The current study used the same line, which divided the surface of the head into anterior and posterior portions.

During rotation at 45° of abduction, the glenoid trajectories extended mainly over the anterior portion of the head. The most proximal part of the shaft axis was present in Zone III of the surface of the head. Thus, the center of the head was located between the glenoid and the shaft on which the rotator cuff and other muscles act during that rotation. In that relationship, the glenoid may play the role of the fulcrum, allowing the upper extremity to easily move in front of the trunk. Although the humerus was positioned in the same relationship to the trunk, the joint has multiple axes with varying angles of the humerus to the glenoid, as shown by the large standard deviation in the angle of horizontal abduction. When the arm is elevated from the dependent position, the insertion of the rotator cuff muscles approaches the glenoid surface. The glenoid trajectory range becomes limited, converging to the top of the head. However, the manner in which this range became limited was not uniform. In fact, during rotation at 90° of abduction, the glenoid trajectories extended over the posterior portion of the head. In this posterior head portion of the head, we could extend the arm toward the backside of the trunk.

When the arm was highly elevated at 135° of abduction, the glenoid trajectories were more limited than those during rotation at lower abductions. The shaft was stabilized by the shortened rotator cuff on both sides. Glenohumeral relationships in highly elevated positions would be more suitable for supporting the arm and weight held by the arm than for mobility with the glenoid playing the role of the platform. The humerus could pivot against the glenoid, and the angles of axial rotation would still be maintained with the shaft axis tilting in the same posterior–inferior direction. Rotation using the glenoid as the platform would help to generate or preserve rotational torque. Throwing athletes such as baseball players or javelin throwers, who must accelerate their arm rotation while playing their sport, might use the glenohumeral joint in the above-described relationship.

As a clinical relevance, information in the current study would be useful for thinking about glenohumeral instability. Gagey et al. [4] described the importance of the inferior glenohumeral ligament for determining the range of movement, especially when the arm is elevated to the preferential position from the coronal plane. Their description suggested the ligament played an important role to determine the glenoid trajectories converging to the top of the head from posterior portion of the head. A Hill–Sachs lesion is a common injury associated with anterior glenohumeral instability. This lesion is about the area of the humeral head in contact with the glenoid at 135° of abduction, when external rotation is performed. If there existed a Bankart lesion causing insufficient function of the ligament, the glenoid trajectory might extend more posteriorly to engage a Hill–Sachs lesion. Thus, a defect which is large or combined with a Bankart lesion, would play the harmful role in anterior joint dislocation or instability and this might be the reason why the joint shows instability when the arm is in highly elevated positions essentially suitable for supporting the arm. As far as the arm is rotated at 45° of abduction, a Hill–Sachs lesion would cause no harm because its lesion stays off the glenoid trajectory.

Certain shortcomings of this study must be acknowledged. The study was based on only six static positions in a small number of subjects, and each glenoid trajectory connecting the internal and external positions was not analyzed directly during active rotation. The participants had to maintain the same position for scanning for about 10 min, and the actual end range of rotation might be wider than the values obtained in this study. Dynamic studies using a wider range of motion should be performed to supplement the present data and provide a better understanding of shoulder motion. Additionally, each participant had to lie in the supine position in an open MRI system. A system with upright coils should be used to investigate standing or sitting positions and determine the effect of gravity.

Although the current study has some limitations, it shows that the angle of abduction might influence shoulder function through its effects on the portion of the humeral surface in contact with the glenoid and the resultant changes in the glenohumeral relationships during rotation.
